# Impact of Isolation Time of COVID-19 Patients in Intensive Care Unit on Healthcare Workers Contamination and Nursing Care Intensity

**DOI:** 10.3389/fmed.2022.824563

**Published:** 2022-03-25

**Authors:** Denis Doyen, Lucas Morand, Mathieu Jozwiak, Didac Aurenche Mateu, Clément Saccheri, Hervé Hyvernat, Marion Cremoni, Vesna Brglez, Nicolas Bèle, Gilles Bernardin, Barbara Seitz-Polski, Jean Dellamonica

**Affiliations:** ^1^Service de Médecine Intensive Réanimation, Centre Hospitalier Universitaire de Nice, Hôpital Archet 1, Nice, France; ^2^UR2CA – Unité de Recherche Clinique Côte d’Azur, Université Côte d’Azur (UCA), Nice, France; ^3^LP2M – CNRS Laboratoire de Physiomédecine Moléculaire, Université Côte d’Azur (UCA), Nice, France; ^4^Laboratoire d’Immunologie, Centre Hospitalier Universitaire de Nice, Hôpital Archet 1, Nice, France; ^5^Centre Hospitalier Intercommunal de Fréjus Saint-Raphaël, Réanimation Polyvalente, Fréjus, France

**Keywords:** COVID-19, isolation, healthcare workers, contamination, nursing care, adverse events

## Abstract

**Background:**

The optimal isolation time of COVID-19 patients in intensive care unit (ICU) is debated. We investigated the impact of two different COVID-19 patient isolation time strategies on healthcare workers (HCW) contamination, intensity of nursing care and potential associated adverse events.

**Methods:**

We prospectively included all consecutive COVID-19 patients and HCW in our ICU in the first two pandemic waves (March to May 2020 and August to November 2020). Specific isolation measures for COVID-19 patients were released after two negative RT-PCR assays in the first wave and 14 days after the onset of symptoms in the second wave. Contamination of HCW was assessed at the end of each pandemic wave by combining both a RT-PCR assay and a serological test.

**Results:**

Overall, 117 COVID-19 patients and 73 HCW were included. Despite an earlier release from isolation after ICU admission in the second than in the first wave [6 (4–8) vs. 15 (11–19) days, *p* < 0.01], the proportion of HCW with a positive serological test (16 vs. 17%, *p* = 0.94) or with a positive RT-PCR assay (3 vs. 5%, *p* = 0.58) was not different between the two waves. Although a lower nurse-to-bed ratio, the intensity of nursing care was higher in the second than in the first wave. A longer isolation time was associated with accidental extubation (OR = 1.18, 95%CI:1.07–1.35, *p* = 0.005) but neither with ventilator-associated pneumonia nor with dysglycemia.

**Conclusion:**

A shorter isolation time of COVID-19 patients in ICU was not associated with higher HCW contamination, while a longer isolation time seemed to be associated with higher accidental extubation.

## Introduction

From December 2019 in China, a worldwide pandemic with an emergent coronavirus SARS-CoV-2 is responsible for Coronavirus disease (COVID-19) (1). As with all respiratory virus outbreaks, isolation of COVID-19 patients is one of the most important precautions to protect healthcare workers (HCW) from SARS-CoV-2 contamination, particularly in intensive care unit (ICU) (2, 3). However, isolation may be harmful to the patient by reducing the intensity of nursing care, which can potentially lead to adverse events (4). So, the optimal isolation time of COVID-19 patients is still under debate (2, 5, 6).

In our ICU, two different COVID-19 patient isolation time strategies were used in the first two pandemic waves. In the first wave (March to May 2020), isolation was released only after two negative real-time reverse transcriptase-polymerase chain reaction (RT-PCR) assays, according to the World Health Organization recommendations (2). In the second wave (August to November 2020), isolation was released 14 days after the onset of symptoms, regardless of the date of hospitalization, according to the French recommendations (5).

Here we investigated the impact of these two COVID-19 patient isolation time strategies on HCW contamination, intensity of nursing care and the potential associated adverse events.

## Patients and Methods

### Patients and Healthcare Workers

This single-center and prospective study was conducted in the 13-bed ICU of Nice university hospital. The study was reviewed and approved by our local institutional review committee (NCT number: NCT04355351; IDRCB number: 2020-A00908-31). All patients or next of kin and all HCW were informed about the study and consented to participate.

We included all consecutive COVID-19 patients admitted in our ICU in the first two pandemic waves (March to May 2020 and August to November 2020) with positive RT-PCR assay in nasal swabs or pulmonary samples. There were no exclusion criteria.

We included all consecutive HCW working in our ICU in the first two pandemic waves. Exclusion criteria were the following: (i) refusal to perform a serological test and a RT-PCR assay at the end of the pandemic waves, (ii) prior confirmed SARS-CoV-2 infection and (iii) HCW already included in the first wave.

### Isolation Protocol of COVID-19 Patients

All COVID-19 patients were hospitalized in well-ventilated single rooms and the doors were kept closed. Transporting patients out of their room was avoided, except in cases of medical or surgical necessity. During the transport, the non-intubated patients were required to always wear a surgical mask (7–9).

A dedicated and limited HCW team has been designated for the management of COVID-19 patients and the number of HCW entering the patient room was reduced to the bare minimum required for patient care. All HCW practiced hand hygiene before putting on and after removing their personal protective equipment, which was donned outside the patient room, before opening the door. The protective equipment consisted in a medical mask FFP2, mobcap, eye protection or facial protection to avoid contamination of mucous membranes, non-sterile long-sleeved gown and medical gloves. For all aerosol-generating procedures, in addition to the protective personal equipment described above, HCW were also required to wear a waterproof apron. HCW used the same protective equipment when transporting patients. Outside the patient room, HCW were required to always wear a surgical mask and were advised not to touch their eyes, nose or mouth with their gloved or bare hands that might be contaminated. Finally, all surfaces were frequently cleaned and disinfected (7–9).

These specific isolation measures for COVID-19 patients were released only after two negative RT-PCR assays in the first wave and 14 days after the onset of symptoms, regardless of the date of hospitalization in the second wave. No viral cultures were performed prior to the release of specific isolation measures, even in immunocompromised patients. Afterward, the same standard precautions were applied in all COVID-19 patients during both pandemic waves: the door of patient room was always opened, gloves, eyes protections and mobcap were removed and HCW wore a surgical mask instead of a FFP2 and waterproof aprons instead of non-sterile long-sleeved gowns (7–9). Thus, only the isolation time differed between the two pandemic waves.

### Assessment of Healthcare Workers Contamination

The HCW contamination was assessed at the end of each pandemic wave by combining both a RT-PCR assay and a serological test. The diagnosis of recent SARS-CoV-2 infection was based on a positive serological test (IgA or both IgA and IgG) and/or a positive RT-PCR assay. Vaccination against COVID-19 had not yet started during the study period.

Two sets of RT-PCR in nasal swabs were used: either Allplex^®^ 2019-nCoV assay (Seegene, Seoul, South Korea) with Microlab NIMBUS^®^ extractor (Hamilton Bonaduz AG, Rapperswil-Jona, Switzerland) and CFX96 thermocycler (Bio-Rad laboratories, Hercules, California, United States of America); or RealStar^®^ SARS-CoV-2 RT-PCR Kit 1.0 assay (Altona Diagnostics, Hamburg, Germany) with QIAsymphony SP^®^ extractor (QIAGEN, Hilden, Germany) and QuantStudio^®^ thermocycler (Thermo Fisher Scientific, Waltham, Massachusetts, United States of America).

Serological tests for anti-SARS-CoV-2 IgA and IgG isotype antibodies were performed with a commercially available enzyme-linked immunosorbent assays (ELISA), which used the S1-domain of the spike protein of SARS-CoV-2 as the antigen (Euroimmun AG, Lübeck, Germany, # EI 2606-9601 A and # EI 2606-9601 G). They were run on a IF Sprinter IFT/ELISA (Euroimmun) according to the manufacturer’s protocol. The results were evaluated by calculating the ratio between the optical density (OD) of the sample at 450 nm and the OD of the calibrator at 450 nm, according to the following formula: OD ratio = OD of the sample/OD of the calibrator. According to the manufacturer’s recommendations, the results were then interpreted as follows: negative (OD ratio < 0.8 for both IgA and IgG), indeterminate (0.8 ≤ OD ratio < 1.1 for IgA and/or IgG) or positive (OD ratio ≥ 1.1 for IgA and/or IgG).

### Assessment of Nursing Care Intensity and Associated Adverse Events

Nursing care intensity was assessed by calculating the OMEGA score (10) and by recording the daily number of the following procedures, that are considered a standard of care in our ICU: Behavioral Pain Scale evaluation, blood glucose measurement, bed-bath as well as oral, eye and intubation care.

According to the Iatroref III study (11), we also recorded the following potential associated adverse events: accidental extubation, hypoglycemia, hyperglycemia, and ventilator-associated pneumonia.

All these procedures and adverse events were prospectively and daily recorded by the nurses and intensivists in the patients’ electronic medical record (Metavision iMDsoft^®^, Tel Aviv, Israel).

### Statistical Analysis

Shapiro-Wilk test was used to assess the distribution of each variable. Continuous variables were summarized as mean ± standard deviation or median [interquartile range] as appropriate and categorical variables were summarized as counts and percentages. Categorical variables were compared with Fisher exact or chi-2 tests and continuous variables were compared with Student-t or Mann-Whitney U tests. The association between the different associated adverse events and the isolation time were assessed using logistic regression, by calculating the unadjusted odds ratio (OR) and their 95% confidence interval [95%CI]. A *p*-value < 0.05 was considered statistically significant. Statistical analyses were performed using the Statistical Package for Social Science software 19 (SPSS, Chicago, Illinois, United States).

## Results

### Study Population

In the two pandemic waves, 117 COVID-19 patients were admitted in our ICU: 41 in the first wave and 76 in the second wave. Overall, 89 (76%) patients were men with a median age of 69 [59–74] years old and a median body mass index of 28 [26–31] kg/m^2^, and 18 (16%) had immunocompromised status. Among immunocompromised patients, 10 (55%) were considered severely immunocompromised: 6 were receiving immunosuppressive treatments, 3 had a medical history of chronic autoimmune disease, and 4 had active cancer or recent chemotherapy (<6 months). The ICU mortality rate was 16%. In the second wave, patients had more chronic arterial hypertension and received more corticosteroids or immunomodulating agents than in the first wave (Table 1). Other patient characteristics and outcomes in both waves are shown in Table 1.

**TABLE 1:** Patient characteristics and outcomes in the two pandemic waves of COVID-19.

	First wave (*n* = 41)	Second wave (*n* = 76)	*P* value
**Characteristics**			
Age, years	65 [53–73]	70 [61–74]	0.07
Male sex, no. (%)	34 (83)	55 (72)	0.20
Body mass index, median, kg/m^2^	28 ± 4	28 ± 6	0.88
Simplified Acute Physiology Score II,	40 ± 18	38 ± 11	0.58
SOFA score at ICU admission,	3 [2–8]	3 [2–6]	0.75
**Comorbidities**			
Obesity, no. (%)	14 (34)	24 (32)	0.78
Chronic arterial hypertension, no. (%)	13 (32)	41 (54)	0.02
Diabetes mellitus, no. (%)	9 (22)	25 (33)	0.21
Cardiovascular disease, no. (%)	4 (10)	8 (11)	0.88
Chronic respiratory disease, no. (%)	4 (10)	7 (9)	0.92
Chronic renal disease, no. (%)	3 (7)	10 (13)	0.34
Immunocompromised status, no. (%)	6 (15)	12 (16)	0.85
**Treatment administered during ICU stay**			
Glucocorticoids, no. (%)	29 (71)	75 (99)	< 0.01
Hydrocortisone, no. (%)	16 (39)	0 (0)	< 0.01
Dexamethasone, no. (%)	0 (0)	75 (98)	< 0.01
Methylprednisolone, no. (%)	13 (32)	0 (0)	< 0.01
Other immunomodulating agents, no. (%)	5 (12)	23 (30)	0.03
Intermediate or full-dose thromboprophylaxis, no. (%)	26 (63)	74 (97)	< 0.01
Antibiotics, no. (%)	35 (85)	58 (76)	0.25
**Respiratory support**			
Invasive mechanical ventilation, no. (%)	28 (68)	40 (53)	0.10
Non-invasive ventilation, no. (%)	0 (0)	0 (0)	1.00
High-flow oxygen therapy, no. (%)	14 (34)	36 (47)	0.17
**Outcomes**			
Duration of invasive mechanical ventilation, days	19 [9–30]	7 [4–16]	< 0.01
ICU length of stay, days	15 [7–27]	8 [5–13]	0.01
ICU mortality, no. (%)	12 (29)	7 (9)	< 0.01

*n = 117, variables are expressed as numbers (%), mean ± standard deviation or median [interquartile range]. no., number of patients; COVID-19, coronavirus disease 2019; ICU, intensive care unit; SOFA, Sepsis-related Organ Failure Assessment.*

**FIGURE 1:**
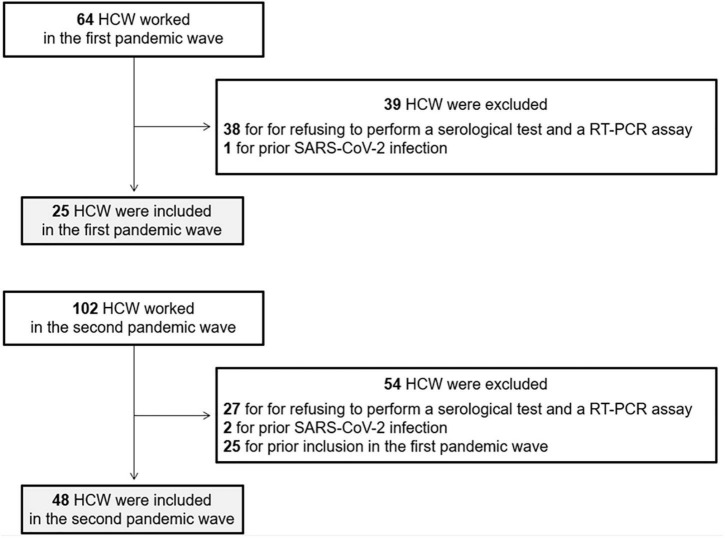
Flowchart of healthcare workers (HCW) inclusion.

Among the 166 HCW working in our ICU in the first two pandemic waves, 73 were included: 25 in the first wave and 48 in the second wave, representing 39 and 47% of HCW, respectively. Sixty-five (70%) HCW were excluded for refusing to perform a serological test and a RT-PCR assay, three (3%) HCW for prior SARS-CoV-2 infection and 25 (27%) for prior inclusion in the first wave (Figure 1). Overall, 30 (41%) HCW were men with a mean age of 40 ± 11 years old. There were more HCW men in the first wave than in the second wave (64 vs. 29%, p < 0.01) but age of HCW was not different between the two waves. No HCW were immunocompromised, as all HCW at risk of developing a severe form of SARS-CoV-2 infection stopped working during the pandemic.

### Healthcare Workers Contamination

**FIGURE 2:**
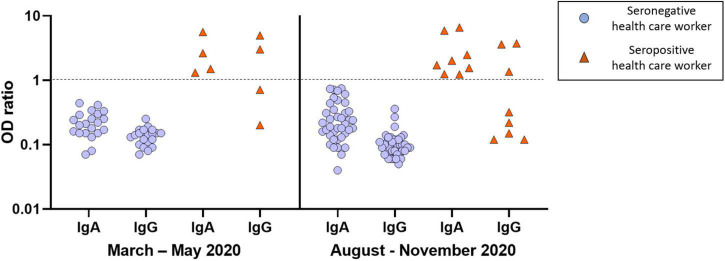
Results of serological tests of healthcare workers in the first (March to May 2020) and second (August to November 2020) pandemic wave. Each healthcare worker was represented by a pair of IgA and IgG symbols. Red triangles represent healthcare workers with positive serological test (*n* = 12: four in the first wave and eight in the second wave). Blue circles represent healthcare workers with negative serological test (*n* = 61: 21 in the first wave and 40 in the second wave). A log-10 scale was used for the *Y* axis [log10 of OD (optical density) ratio]. The dotted line represents the threshold value of positivity. The results were interpreted as follows: negative: OD ratio < 0.8 for both IgA and IgG; indeterminate: 0.8 ≤ OD ratio < 1.1 for IgA and/or IgG; positive: OD ratio ≥ 1.1 for IgA and/or IgG.

The isolation protocol implemented in our ICU has been adhered by all HCW and compliance with the isolation protocol was the same during both waves of the pandemic. Despite an earlier release from isolation after ICU admission in the second wave than in the first wave [6 (4–8) vs. 15 (11–19) days, *p* < 0.01], the proportion of HCW with a positive serological test (16 vs. 17%, *p* = 0.94) or with a positive RT-PCR assay (3 vs. 5%, *p* = 0.58) was not different between the two waves (Figure 2). All HCW with a positive RT-PCR assay had a positive serological test and no HCW had an indeterminate serological test.

Overall, 12 (16%) HCW developed a SARS-CoV-2 infection: four in the first wave and eight in the second wave. Among them, none had severe infection, 10 (83%) were mildly symptomatic and 2 (17%) were asymptomatic. SARS-CoV-2 infection was diagnosed only on a positive serological test in 8 (67%) HCW and on both a positive serological test and RT-PCR assay in 4 (33%) HCW. Among HCW with a positive serological test, seven (58%) had IgA only, five (42%) had both IgA and IgG and none had IgG only. The proportion of HCW with IgA only (50 vs. 62%, *p* = 1.00, respectively) or with both IgA and IgG (50 vs. 38%, *p* = 1.00, respectively) was not different between the two waves.

### Nursing Care Intensity and Associated Adverse Events

Although a lower nurse-to-bed ratio, the intensity of nursing care was higher in the second wave than in the first [17 (14–22) vs.14 (12–17), *p* < 0.01] with more daily BPS evaluations and daily blood glucose measurements (Table 2). The OMEGA score was not different between the first and the second wave [94 (31–153) vs. 168 (28–390), *p* = 0.27].

**TABLE 2:** Patient management in the two pandemic waves.

	First wave (*n* = 41)	Second wave (*n* = 76)	*P* value
Isolation time in ICU, days	15 [11–19]	6 [4–8]	<0.01
Isolation time in ICU, percentage of ICU length of stay^a^	100 [72–100]	57 [28–100]	<0.01
**Intensity of nursing care**			
Nurse-to-bed ratio	0.6 [0.5–0.6]	0.5 [0.4–0.5]	<0.01
OMEGA score	94 [31–153]	168 [28–390]	0.27
Number of nursing care, per day	14 [12–17]	17 [14–22]	<0.01
Number of Behavioral Pain Scale evaluation, per day	2 [1–4]	5 [3–7]	0.02
Number of blood glucose measurement, per day	6 [5–9]	9 [8–10]	<0.01
Number of bed-bath, per day	2 [1–2]	2 [1–2]	0.75
Number of oral care, per day of invasive mechanical ventilation^b^	2 [2–3]	3 [2–3]	0.74
Number of eye care, per day of invasive mechanical ventilation^b^	2 [2–3]	2 [2–3]	0.52
Number of intubation care, per day of invasive mechanical ventilation^b^	1 [1–2]	1 [1–1]	0.07
**Adverse events**			
Accidental extubation^b^, no. (%)	11 (39)	4 (10)	<0.01
Ventilator-associated pneumonia^b^, no. (%)	12 (43)	23 (58)	0.12
Hypoglycemia^c^, no. (%)	23 (56)	31 (43)	0.16
Hyperglycemia^c^, no. (%)	39 (96)	72 (98)	0.38

*n = 117, variables are expressed as numbers (%) or median [interquartile range]. no., number of patients; ICU, Intensive Care Unit. ^a^Isolation time/ICU length of stay ×100. ^b^In patients under invasive mechanical ventilation (n = 28 in the first wave and n = 40 in the second wave). ^c^Defined as blood glucose ≤ 0.8 g/L and > 1.4 g/L, respectively.*

In logistic regression, a longer isolation time was associated with accidental extubation (OR = 1.18, 95%CI:1.07–1.35, *p* = 0.005) but neither with ventilator-associated pneumonia (OR = 1.12, 95%CI:1.02–1.24, *p* = 0.07) nor with hyperglycemia (OR = 0.99, 95CI%:0.84–1.34, *p* = 0.72) or hypoglycemia (OR = 1.04, 95CI%:0.97–1.11, *p* = 0.30). After adjustment to the duration of invasive mechanical ventilation, a longer isolation time was no longer associated with accidental extubation (OR = 0.99, 95%CI:0.98–1.01, *p* = 0.91).

## Discussion

While isolation of COVID-19 patients is one of the most important precautions to limit the spread of the virus and thus to protect HCW from SARS-CoV-2 contamination, the optimal isolation time in these patients is still debated. Releasing isolation too early may lead to HCW contamination while too long isolation time could impact COVID-19 patient management. To our knowledge, this is the first study evaluating the impact of isolation time of critically ill COVID-19 patients both on HCW contamination and patient management. We found that a shorter isolation time did not lead to an increase in HCW contamination and was associated with a higher intensity of nursing care despite a lower nurse-to-bed ratio. Conversely, a longer isolation time was associated with accidental extubation while this association was no longer found after adjustment to the duration of invasive mechanical ventilation.

Healthcare workers contamination did not increase between the two pandemic waves, while release from isolation after ICU admission was significantly earlier in the second than in the first wave despite a longer duration of exposure of HCW to SARS-CoV-2 in the second wave (4 vs. 3 months). Our results suggest that the risk of HCW contamination is shorter than that predicted by RT-PCR and that guiding the duration of isolation of COVID-19 patients on RT-PCR may lead to excessive isolation times. This could be explained by the fact that even in critically ill COVID-19 patients with higher viral loads (2, 3), SARS-CoV-2 clearance assessed by culture may be quicker than that assessed by RT-PCR (2, 12). Interestingly, HCW contamination was assessed by combining both serological tests and RT-PCR assays. Since we previously found that 45% of HCW had asymptomatic forms of COVID-19 (13), we decided to combine both serological tests and RT-PCR assays to ensure that all COVID-19 contaminations, including HCW with asymptomatic COVID-19 forms, would be diagnosed. For this purpose, we used an IgA serological test to identify very early forms of COVID-19, i.e., within one month of contamination (14, 15). It is of importance to note that no HCW had IgG only and more than half of HCW had IgA only, highlighting the fact that HCW contamination was recent. It cannot be excluded that some HCW were contamined outside the ICU. Nevertheless, the incidence of HCW contamination is much higher among HCW than in the general population (13), suggesting that HCW contamination we observed in our cohort occurred primarily in ICU.

Despite a lower nurse-to-bed ratio, we found that a shorter isolation time was associated with a higher intensity of nursing care, illustrated by more daily BPS evaluations and blood glucose measurements. However, the OMEGA score was not different between the two waves. This discrepancy could be explained by the fact that the OMEGA score, unlike other scores such as the NAS score (16) used to assess nurses ’activities and workload, also includes some items assessing medical activities (10). Conversely, a longer isolation time was associated with more accidental extubation but neither with ventilator-associated pneumonia nor with dysglycemia. However, after adjusting to the duration of invasive mechanical ventilation, the isolation time was no longer associated with accidental extubation. Our results were not in agreement with those by Zahar et al., who showed that contact isolation in patients isolated for multidrug-resistant organisms was associated with more accidental extubation, less strict glucose control and more ventilator-associated pneumonia (4). We cannot exclude a lack of power to explain this discrepancy.

Our study has some limitations. First, there was a limited sample size of patients and less than half of HCW involved in COVID-19 patients management were included. However, HCW contamination was assessed by combining both serological tests and RT-PCR assays and COVID-19 patients had similar characteristics and severity in both waves, making relevant the comparison. In addition, the single-center design implied that the isolation protocol of COVID-19 patients and the standard precautions were exactly the same between both pandemic waves. Second, our results are not generalizable to new emergent SARS-CoV-2 variants and to patients with profound immunosuppression, who may shed viable SARS-CoV-2 for a few weeks (17). In these patients, it may be necessary to extend the period of isolation to 20 days after the onset of symptoms and a test-based strategy to determine the appropriate duration of isolation may be of interest, as recommended by the Centers for Disease Control and Prevention (18).

To conclude, a shorter isolation time of COVID-19 patients in ICU was not associated with higher HCW contamination but with higher intensity of nursing care. Conversely, a longer isolation time seemed to be associated with higher accidental extubation. Further studies are needed to confirm the potential impact of isolation time of critically ill COVID-19 patients.

## Data Availability Statement

The data analyzed in this study is subject to the following licenses/restrictions. The dataset used and analyzed for the current study is available from the corresponding author on reasonable request. Requests to access these datasets should be directed to DD, doyen.d@chu-nice.fr.

## Ethics Statement

Ethical review and approval was not required for the study on human participants in accordance with the local legislation and institutional requirements. The patients/participants provided their written informed consent to participate in this study.

## Author Contributions

DD, LM, DA, CS, HH, MC, VB, and BS-P recorded the data. DD, LM, MJ, BS-P, and JD drafted the first version of the manuscript. All authors conceived the study, analyzed and interpreted the data, and read and approved the final manuscript.

## Conflict of Interest

The authors declare that the research was conducted in the absence of any commercial or financial relationships that could be construed as a potential conflict of interest.

## Publisher’s Note

All claims expressed in this article are solely those of the authors and do not necessarily represent those of their affiliated organizations, or those of the publisher, the editors and the reviewers. Any product that may be evaluated in this article, or claim that may be made by its manufacturer, is not guaranteed or endorsed by the publisher.

## Abbreviations

COVID-19: coronavirus disease
HCW: healthcare workers
ICU: intensive care unit
OD: optical density
RT-PCR: reverse transcriptase-polymerase chain reaction.
